# Absolute and Relative Agreement between the Current and Modified Brazilian Cardioprotective Nutritional Program Dietary Index (BALANCE DI) and the American Heart Association Healthy Diet Score (AHA-DS) in Post Myocardial Infarction Patients

**DOI:** 10.3390/nu14071378

**Published:** 2022-03-25

**Authors:** Camila Weschenfelder, Philip Sapp, Terrence Riley, Kristina Petersen, Jacqueline Tereza da Silva, Angela Cristine Bersch-Ferreira, Rachel Helena Vieira Machado, Erlon Oliveira de Abreu-Silva, Lucas Ribeiro Silva, Bernardete Weber, Alexandre Schaan de Quadros, Penny Kris-Etherton, Aline Marcadenti

**Affiliations:** 1 Graduate Program in Health Sciences (Cardiology), Instituto de Cardiologia do Rio Grande do Sul, Fundação Universitária de Cardiologia (IC/FUC), Princesa Isabel Avenue 395, Porto Alegre 90040-371, Brazil; camilawesche@gmail.com (C.W.); consult.asq@gmail.com (A.S.d.Q.); 2 Department of Nutritional Sciences, Pennsylvania State University, 110 Chandlee Laboratory, University Park, State College, PA 16802, USA; philip.sapp@psu.edu (P.S.); tmr359@psu.edu (T.R.); pmk3@psu.edu (P.K.-E.); 3 Department of Nutritional Sciences, Texas Tech University, Lubbock, TX 79409, USA; kristina.petersen@ttu.edu; 4 Global Academy of Agriculture and Food Security, University of Edinburgh, Sir Alexander Robertson Building, Easter Bush Campus, Edinburgh EH25 9RG, UK; jacqueline.silva@ed.ac.uk; 5 Hcor Research Institute, Hcor (IP-Hcor), Abílio Soares Street 250, São Paulo 04004-050, Brazil; abferreira@hcor.com.br (A.C.B.-F.); rhelena@hcor.com.br (R.H.V.M.); erlon@terra.com.br (E.O.d.A.-S.); lucribeiro@hcor.com.br (L.R.S.); bweber@hcor.com.br (B.W.)

**Keywords:** dietary patterns, diet, healthy, myocardial infarction

## Abstract

The American Heart Association Diet Score (AHA-DS) defines the cardiovascular health, and the Brazilian Cardioprotective Nutritional Program Dietary Index (BALANCE DI) was designed to evaluate diet quality in secondary cardiovascular prevention settings. Our aim was to assess the absolute and relative agreement between both tools in Brazilian adults after a myocardial infarction (MI). In this cross-sectional study, 473 individuals were included and had their diet assessed by a 24 h food recall and a semi-quantitative Food Frequency Questionnaire. The weighted Kappa between BALANCE DI and primary AHA-DS was 0.66 (95% CI: 0.08–0.21), and between BALANCE DI and total AHA-DS was 0.70 (95% CI: 0.20–0.32). To improve the agreement between the tools, modifications were made to the BALANCE DI scoring system. The weighted Kappa between New BALANCE DI and primary AHA-DS was 0.77 (95% CI: 0.36–0.48), and between BALANCE DI and total AHA-DS was 0.76 (95% CI: 0.34–0.46). The mean bias observed between the New BALANCE DI as compared to the primary and total AHA-DS was −16% (−51 to 19) and −8% (−41 to 24), respectively. Our results suggest that the New BALANCE DI may be a useful tool to evaluate diet quality in post MI patients.

## 1. Introduction

Ischemic heart disease (IHD) is the leading cause of death worldwide [1]. Myocardial infarction (MI) is a common manifestation of IHD and is the greatest cause of cardiovascular mortality in Brazil [2]. Diet is a modifiable risk factor for IHD [3,4]. Many epidemiologic studies and clinical trials have shown that a healthy dietary pattern is associated with primary and secondary prevention of cardiovascular disease (CVD) [5,6,7,8]. This evidence is the basis of current dietary recommendations for the prevention and treatment of CVD [9,10].

In the 2020 American Heart Association (AHA) Impact Goal, a healthy eating pattern consistent with the DASH (Dietary Approaches to Stop Hypertension) diet was recommended for cardiovascular health (CVH) [11]. This healthy eating pattern is the basis for the scoring system developed by the AHA to evaluate diet quality and define CVH. The AHA proposed dietary targets and a healthy diet score (AHA-DS) [12], which classifies the dietary pattern as ideal, intermediate, or poor. Few studies have evaluated the AHA-DS in patients after an MI [13].

The Brazilian Cardioprotective Nutritional Program (BALANCE) is a regional and feasible dietary pattern that was designed to improve diet quality in adults with atherosclerotic cardiovascular disease [14,15,16]. BALANCE is composed of four food groups represented by the three colors of the Brazilian flag (green, yellow, and blue represent recommended foods and red represents foods to avoid) [15]. To assess adherence to the BALANCE recommendations, the BALANCE DI was developed [17]. Thus, the BALANCE DI reflects adherence to a country-specific recommended dietary pattern. The BALANCE DI performs similarly to other diet quality indices regarding reliability and construct validity [17]. 

Although it can be used to assess diet quality in adults with IHD, it is unknown how BALANCE DI relates to CVH. This study was conducted to assess the absolute and relative agreement between the BALANCE DI and the AHA-DS in Brazilian adults after an MI (2 to 6 months after the event). Investigation of this country-specific diet quality index is needed because cultural adaption of recommended dietary patterns to the target population enhances adherence. 

## 2. Materials and Methods

### 2.1. Study Design and Participants

This is a cross-sectional analysis of baseline data from the DICA-NUTS Study, for which a detailed protocol was previously published [18]. Briefly, DICA-NUTS is a 16-week, parallel, multicenter, randomized clinical trial (ClinicalTrials.gov NCT03728127) carried out in four regions of Brazil (Northeast, Southeast, South, and Midwest), from January 2019 to December 2021 [18]. The study included patients over 40 years old with diagnosed MI, either ST-Elevation MI (STEMI) or non-STEMI in the last 2 to 6 months. At baseline, participants completed questionnaires on sociodemographic, lifestyle, medical, and dietary intake. All data were collected by trained nutritionists [18]. Ethical approval for the analysis was provided by Research Ethics Committee of Instituto de Cardiologia do Rio Grande do Sul/Fundação Universitária de Cardiologia (IC/FUC) under number 5.115.455 (CAAE 52734921.0.0000.5333). All participants provided written informed consent.

### 2.2. Dietary Assessment

At baseline, participants completed one 24-h food recall (24hR) and a semi-quantitative Food Frequency Questionnaire (FFQ) [18]. Both tools were administered on the same day during the participant’s first appointment. The FFQ collected data on their consumption over the previous 365 days. A photo album with standardized household measures or grams was used in both the 24hR and FFQ [18,19]. For the analysis of the 24hR, a computerized system (Sistema Vivanda de Alimentação^®^, São Paulo, Brazil) was used that prioritizes Brazilian nutrition composition tables [18].

#### 2.2.1. BALANCE DI

The BALANCE DI was calculated based on the 24hR. The scoring system considers the four BALANCE food groups [17] (Supplemental Table S1). Foods are classified into each food group using the BALANCE recommendations tool that categorizes foods by caloric equivalents and the density of sodium, saturated fat, and cholesterol. For each food group, portions consumed were summed and scores were determined for individuals based on energy intake from the 24hR. The caloric ranges and recommended intake of food groups are described in Supplemental Table S2 [18]. 

Supplemental Table S3 summarizes the BALANCE DI score. Higher scores represent greater adherence to recommendations [17]. The scores were calculated as continuous variables and rounded to the nearest tenth decimal point. 

#### 2.2.2. AHA-DS

The AHA-DS for ideal, intermediate, and poor dietary patterns uses a binary scoring system [12]. The highest score of 10 is given for meeting or exceeding the AHA target, and the lowest score of zero is given for no intake of the following cardioprotective dietary factors, i.e., fruits and vegetables, fish and shellfish, whole grains, nuts, seeds, and legumes, or for high intake of foods/nutrients that should be limited (i.e., sodium, sugar-sweetened beverages, processed meats and saturated fat). Scores are determined on a continuous scale (rounded to the nearest whole number). The range of the primary AHA-DS is 0 to 50 (for 5 components: fruits and vegetables, fish and shellfish, whole grains, sodium, and sugar-sweetened beverages). 

Three secondary components (nuts, seeds, and legumes; processed meats; and saturated fat) are included in the total AHA-DS. The range for the total AHA-DS is 0 to 80 (for 8 components: the 5 components for the primary score + the 3 components for the secondary score). For both the primary AHA-DS and the total AHA-DS, an ideal score is given for meeting ≥80% of the targets, an intermediate score corresponds to 40–79% of the targets, and a poor score corresponds to meeting <40% of the targets. The AHA scoring system is summarized in Supplemental Table S4.

AHA scores were determined using the available data from the FFQ and the 24hR. Data from the 24hR were used for components scored as servings per day (fruits and vegetables, whole grains, saturated fat, and sodium). Scores reported in servings per week (fish and shellfish, sugar sweetened beverages, nuts, seeds, legumes, and processed meats) were derived from the FFQ (in grams or mL). For legumes, reported intake in grams was transformed into cups/day according to the portions defined in the United States Department of Agriculture (USDA) FoodData Central database [20]. All mixed composition foods were scored using estimates of the amount contained within a mixed food item (i.e., the meat content of a hamburger was considered separately from the whole hamburger, including the bun and condiments). A full list of FFQ items and classifications can be found in the Supplemental Tables S5 and S6.

### 2.3. Sociodemographic, Clinic, Lifestyle Variables and Biochemical Assessment

Self-reported sociodemographic data were collected including: sex (male or female), age (years), marital status (single, married, divorced, widowed, stable union), education level (illiterate/incomplete elementary school, complete elementary school/incomplete middle school, complete middle school/incomplete high school, complete high school school/incomplete college degree, complete college degree) [18]. Clinical and lifestyle variables were smoking status (never smoked, ex-smoker, smoker) and comorbidities as determined by previous medical diagnosis such as Type 2 Diabetes Mellitus (T2DM), arterial hypertension, and dyslipidemia [18]. 

Anthropometrics were collected by a licensed nutritionist trained in study procedures as previously described [18]. Parameters collected were: weight (kg), height (m), waist circumference (WC (cm)), and body mass index (BMI (kg/m^2^)). Physical activity was accessed in minutes per week using the International Physical Activity Questionnaire (IPAQ) [18,21].

Fasting blood samples were collected at the baseline visit. Plasma was analyzed for total cholesterol (TC (mmol/L)), high density lipoprotein cholesterol (HDL-C (mmol/L)), triglycerides (TG (mmol/L)), fasting glucose (FG (mmol/L)), fasting insulin (FI (mU/L)), glycated hemoglobin (HbA1c (%)), at the clinical analysis laboratories at each center using standardized techniques [18]. Additionally, low-density lipoprotein (LDL-C (mmol/L)) was calculated from Martin’s mathematical formula [22].

### 2.4. Statistical Analysis

Statistical analyses were performed using SAS (version 9.4; SAS Institute, Cary, NC, USA). All variables were tested for normality (PROC UNIVARIATE) based on the distribution, normal probability plots (Q-Q plots), and skewness. For non-parametric data, natural log transformations were made prior to analysis. To enable direct comparison between the AHA-DS and the BALANCE DI, data from both indices were converted to a percentage of total score for each subject. Paired t-tests were used to assess mean bias of the BALANCE DI compared to the AHA-DS (standard reference). Pearson’s correlations were used to assess the correlation between the two indices. Bland–Altman plots and weighted kappa were used to assess the agreement between the two indices. *p* < 0.05 was considered statistically significant.

## 3. Results

From 486 individuals included in the DICA-NUTS study, a total of 473 were included in this analysis; 13 patients were excluded from the analysis due to missing dietary data at the baseline visit. The average age was 59 ± 9.4 years and 72% were male. The mean BMI was 28.5 ± 4.3 kg/m^2^. At the baseline visit, lipid parameters were within normal limits. The average blood glucose was 6.41 mmol/L (±2.46 mmol/L), HbA1c was 6.33% (±1.4%), and insulin was within normal limits. Table 1 summarizes the characteristics of the study participants.

Diet quality was assessed 108 days ± 36.3 days following MI. Dietary intake according to the 24hR is described in Table 1. Mean energy intake was 1616 ± 624 kcals and sodium intake was 3131.8 ± 1656 mg. Adherence to the primary and total AHA-DS was 59 ± 17% and 51 ± 14%, respectively, which is consistent with an intermediate diet score. Mean adherence to the BALANCE DI was 43 ± 18.7%. 

Comparison between the AHA-DS and the BALANCE DI showed weak to moderate correlations (Table S7). When comparing the BALANCE DI with the primary AHA-DS, 23% of the cohort was ranked in the same quintile by both indices (weighted Kappa was 0.66 (95% CI: 0.08–0.21), Table S8. Partial agreement (±1 quintile) was 24% and gross misclassification (±4 quintiles) was 5.1%. When comparing the BALANCE DI with the total AHA-DS, the exact agreement was 28%, the partial (±1 quintile) was 22% and gross misclassification (±4 quintiles) was 1.5% (weighted Kappa 0.70, 95% CI: 0.20–0.32).

To improve the agreement between the BALANCE DI and the AHA-DS, modifications were made to the BALANCE DI scoring system (Table 2). The modifications we made to the BALANCE DI were to improve the agreement with the AHA-DS components “fruits and vegetables” (extracted from the green group) and “whole grains” (extracted from the yellow group). When both components were extracted from the BALANCE DI green and yellow food groups, the index needed to be rescored and we assumed that 50% needed to be composed of fruits and vegetables and whole grains, respectively. Additionally, when whole grains consumption exceeded the recommendation for the yellow group, a score of 0 was given since one of the principles of the BALANCE DI is energy prescription. The scoring system for the blue and red groups were unchanged.

**Figure 1:**
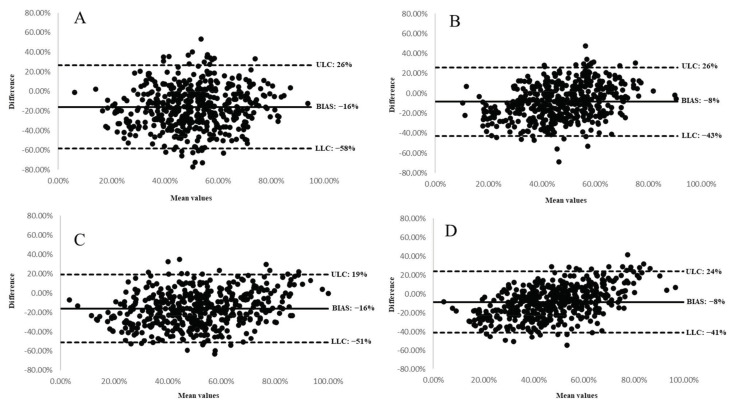
Bland-Altman plots between the BALANCE DI, the New BALANCE DI and both primary and secondary AHA-DS for determining overall diet quality of patients after myocardial infarction (*n* = 473). (**A**) BALANCE DI and primary AHA-DS; (**B**) BALANCE DI and total AHA-DS; (**C**) New BALANCE DI and primary AHA-DS; (**D**) New BALANCE DI and total AHA-DS. The solid line represents the mean difference between the two instruments, and the dotted lines represent the minimum and maximum differences between the scores. ULC: upper limit of concordance; LLC: lower limit of concordance.

The correlations between the New BALANCE DI and the AHA-DS were improved, as shown in Table S9. The New BALANCE DI ranked 35% of the cohort in the same quintile as the AHA-DS (weighted Kappa 0.77 (95% CI: 0.36–0.48). Partial (±1 quintile) was 24% and gross misclassification (±4 quintiles) was 0.6% (Table S10) When comparing the New BALANCE DI with the total AHA-DS, the exact quintile agreement was 36%, the partial quintile agreement (±1 quintile) was 25% and the gross misclassification (±4 quintiles) was 0.4% (weighted Kappa 0.76 (95% CI: 0.34–0.46). Figure 1 shows the Bland–Altman plots for the primary and secondary AHA-DS versus the BALANCE DI (Panel A and B) and the New BALANCE DI (Panel C and D). Proportional bias was observed when comparing the total AHA score with the BALANCE DI (Panel B) and the new BALANCE DI (Panel D) such that bias was greater for those with higher diet quality. 

## 4. Discussion

This study aimed to assess the strength of the relationship and degree of agreement between the BALANCE DI and the AHA-DS in post MI patients in Brazil to evaluate the BALANCE DI as a tool for CVH assessment. The BALANCE DI and the primary AHA-DS were weakly correlated (r = 0.28), which improved modestly when correlating the total AHA-DS (r = 0.46). Similarly, measures of agreement were fair between quintiles of the BALANCE DI and AHA-DS (23%) or the total AHA-DS (28%). Bland–Altman analysis showed that the BALANCE DI was on average higher than both the primary and total AHA-DS with large limits of agreement in both cases. However, some evidence of proportional bias was present whereby bias varied by diet quality.

In general, for dietary index development and/or comparisons, different approaches are used. We used a more comprehensive score, specific for measuring CVH, as a comparator. However, studies that use another tool as the reference for the development of a new index are scarce in the literature. Antonio et al. [23], when comparing the Healthy Eating Index (HEI) and the Diabetes Healthy Eating Index (DHEI) among T2DM patients, reported mean bias of 17 points. These results are similar to ours since comparisons between both the BALANCE and the New BALANCE DI with the primary AHA-DS demonstrate a difference of 16 points. In healthy individuals, comparisons between the agreement of five indexes that measure adherence to a Mediterranean dietary pattern showed a moderate–fair concordance among indexes evaluated by Cohen’s Kappa coefficient, except for the Mediterranean diet score (MDS) and alternative Mediterranean diet (aMED) with a 0.56 (95% CI 0.55–0.59) and 0.67 (95% CI 0.66–0.68) using linear and quadratic weighting, respectively [24]. The authors attributed the disagreement between the indexes to the lack of common criteria to develop the indexes, the type of foods or nutrients considered, the variability of the methods used to construct them, and the dependence or independence of the scores from the study sample, factors that might also explain our results.

Low agreement between the tools may be due to the distinct methods of food classifications between tools. The BALANCE-DI measures adherence to a healthy dietary pattern composed of food groups, that differ from the AHA-DS food groups. For example, the BALANCE DI green group is composed of vegetables, fruits, beans and legumes, and low-fat milk whereas the AHA-DS considers these food groups separately (fruits and vegetables, and legumes are scored as different components). Additionally, in contrast with the AHA-DS, that specifically recommends whole grains, the BALANCE DI yellow group is composed of both refined grains and whole grains.

Modifications to the BALANCE DI focused only on including fruits and vegetables in the green group and removing refined grains from the yellow group improved the correlation with the AHA-DS. However, Bland–Altman analyses showed a wide limit of agreement for the New Balance DI. These results suggest that the New BALANCE DI may be limited in its use for diet assessment of individuals, but may be suitable for use in cohorts of patients post MI. This approach to modifying diet assessment tools may be a useful model for the modification of other culturally specific diet quality assessment tools.

High diet quality has been associated with better prognosis for secondary cardiovascular prevention [25,26]. To evaluate the relationship between diet quality and mortality among MI survivors, Li et al. [25] included 4098 participants that were free of CVD, stroke or cancer at the time of enrollment and survived a first MI during the follow up. Comparing the extreme quintiles of the post-MI Alternative Healthy Eating Index (AHEI) 2010 (excluding the alcohol component), the adjusted HR associated were 0.73 (95% CI: 0.58–0.93) for all-cause mortality and 0.81 (95% CI: 0.64–1.04) for cardiovascular mortality. In a prospective cohort study [26] of 31,546 individuals with prior CVD or DM, higher diet quality was associated with a lower risk of recurrent or new CVD events in people receiving drug therapy for secondary prevention (HR 0.78; 95% CI 0.71–0.87, top versus lowest quintile of modified AHEI; *p* for trend <0.001). The study estimated that at least 20% of CVD recurrence could be avoided by adhering to a healthy diet.

However, few studies have assessed diet quality in patients after an MI, especially with instruments designed specifically for this population. We used a validated Brazilian dietary index to determine adherence to a healthy dietary pattern based on the current national guidelines for secondary CVD prevention [17]. Our study showed relatively poor adherence (43 ± 18.7%) to the BALANCE dietary eating patterns. The association between the BALANCE DI and CVD-related outcomes has not been assessed. The AHA-DS, a validated diet quality assessment tool associated with CVD outcomes, showed that overall diet quality in this sample of 473 post-MI patients was consistent with an intermediate diet score. Previous studies with large samples, but in different populations, reported similar findings. Among 33,932 US [27] and 37,803 Europeans [28], the AHA-DS from both samples was consistent with an intermediate score. Conversely, Mok et al. [13] found that 50.8% of the 1277 participants from the ARIC study (aged 45–64 years old) who developed an MI had a poor diet score [13]. The timepoint at which diet assessment occurs may explain the difference between our findings and those of Mok et al. [13], who collected dietary data prior to the event; we collected data from our sample post-MI. Dietary habits are reportedly altered after a coronary event to align more closely with diet recommendations [29].

A healthy diet is part of The Life’s Simple 7 (LS7) metrics proposed by the AHA for the CVH definition [11]. Besides diet, smoking, physical activity, body mass index, total cholesterol, blood pressure and blood glucose are characterized as being ideal, intermediate, or poor. The achievement of a greater number of ideal metrics is associated with a lower risk of incident CVD [30,31,32,33], including IHD [33], and closer adherence to optimal levels of the 7 CVH metrics is associated with better prognosis after an MI [13]. Thus, the AHA-DS was used in this study for comparison because meeting the AHA recommendations for healthy eating supports cardiovascular risk reduction.

This study has several strengths. We investigated diet quality in patients up to 6 months after an MI and most studies are conducted later. However, the first months after the infarction might be a window of opportunity to improve dietary habits and consequently diet quality. Another strength of our study is the use and modification of a Brazilian DI that measures adherence to a recommended dietary pattern for this population. The New BALANCE DI aligns with the AHA-DS for CVH. Our study has shown that a DI that is based on a culturally unique dietary pattern can be adapted in alignment with the AHA-DS and used to assess diet quality in patients with IHD. Further research is needed to assess the ease of use of the New BALANCE DI in clinical settings.

Our study has several limitations as well. First, the BALANCE DI uses 24hR, whereas our analysis used both the 24hR and the FFQ for scoring the AHA-DS. This was because the AHA-DS considers weekly intake for some components. Thus, our results might reflect differences in the dietary assessment tools used. Second, instead of classifying the AHA-DS components “fruits and vegetables” in cups/day and “whole grains” in oz-equivalent-servings/day, we used the number of portions used for the BALANCE DI scoring, which could have influenced our results, since the BALANCE DI portions are based on caloric equivalents and conversion to household measurements could have resulted in some errors in assessing adherence to current recommendations. Third, the agreement between the BALANCE DI, the New BALANCE DI and the AHA-DS seems to be proportional to diet quality, which is difficult to correct for and suggests additional modifications are needed to the BALANCE DI.

## 5. Conclusions

In post-MI Brazilian adults, the BALANCE DI and the AHA-DS showed limited agreement. Our results suggest that the New BALANCE DI may be used to assess diet quality in cohorts of post MI patients. Further studies are needed to assess the association between the New BALANCE DI with CVH in a primary prevention setting and in patients with established IHD to evaluate its effectiveness in predicting future events.

## Figures and Tables

**Table 1:** Demographic, clinical, lifestyle, laboratory, and dietary intake characteristics of participants (*n* = 473).

Characteristic	Mean ± SD, Median (IQR) or *n* (%)
Age (years)	60 ± 9.4
Sex	
Female	135 (28)
Male	344 (72)
Days after MI	108 ± 36.3
Marital status	
Single	79 (16)
Married	276 (58)
Divorced	59 (12)
Widowed	33 (7)
Common Law Marriage	32 (7)
Education Level	
Illiterate/incomplete elementary school	99 (21)
Complete elementary school/incomplete middle school	87 (18)
Complete middle school/incomplete high school	65 (14)
Complete high school school/incomplete college degree	142 (30)
Complete college degree	84 (17)
Smoking	
No	172 (36)
Ex-smoker	255 (53)
Smoker	50 (11)
T2DM *	134 (28)
Hypertension *	313 (65)
Dyslipidemia *	195 (41)
BMI (kg/m^2^)	28.5 ± 4.3
Weight (kg)	78.2 ± 15.1
WC (cm)	97.9 ± 11.5
WHR	0.9 ± 0.08
Level of Physical Activity (according to IPAQ **)	
High	127 (27)
Moderate	266 (56)
Low	82 (17)
Dietary intake ***	
Energy (Kcal)	1616 ± 624
Carbohydrates (% energy)	52 ± 14
Proteins (% energy)	20 ± 10.1
Fat (% energy)	31 ± 14.2
SFA (% energy)	10.6 ± 5.5
PUFA (% energy)	7.3 ± 4.4
MUFA (% energy)	9.5 ± 5
Dietary Fiber (g)	20.7 ± 8.1
Sodium (mg)	3131 ± 1656
Biochemical data	
TC (mmol/L)	4.08 ± 1.39
LDL-C (mmol/L)	2.2 ± 1.13
HDL-C (mmol/L)	1.09 ± 0.49
TG (mmol/L)	1.5 (0.5–8.39)
Glucose (mmol/L)	6.41 ±2.46
HbA1c (%)	6.33 ±1.4
Insulin (UI/mL)	10.2 (0.1–200)

* Determined by previous medical diagnosis; ** High: vigorous-intensity activity on at least 3 days and accumulating at least 1500 MET-minutes/week OR 7 or more days of any combination of walking, moderate- or vigorous- intensity activities accumulating at least 3000 MET-minutes/week; Moderate: 3 or more days of vigorous-intensity activity of at least 20 min per day OR 5 or more days of moderate-intensity activity and/or walking of at least 3 min per day OR 5 or more days of any combination of walking, moderate-intensity or vigorous-intensity activities achieving a minimum of at least 600 MET-min/week; Low: no activity is reported OR some activity is reported but not enough to meet Categories 2 or 3; *** Based on 24hR. T2DM: Type 2 Diabetes Mellitus; BMI: body mass index; WC: waist circumference; WHR: waist to hip ratio; IPAQ: International Physical Activity Questionnaire; SFA: saturated fatty acids; PUFA: polyunsaturated fatty acids; MUFA: monounsaturated fatty acids; TC: total cholesterol; LDL-C: low density lipoprotein cholesterol; HDL-C: high density lipoprotein cholesterol; TG: triglycerides; HbA1c: glycated hemoglobin.

**Table 2:** Scoring system for the New BALANCE DI.

BALANCE Groups	Recommendation (Portions/d)	Energy Requirements (kcal/d)					
		1400	1600	1800	2000	2200	2400
Green	Original	9	11	11	12	14	16
	Fruits and vegetables	4.5	5.5	5.5	6	7	8
	*Proportionally scored*	0–4.5	0–5.5	0–5.5	0–6	0–7	0–8
	*Score 10*	>4.5	>5.5	>5.5	>6	>7	>8
Yellow	Original	6	7	9	10	11	13
	Whole grains	3	3.5	4	5	5.5	6.5
	*Proportionally scored*	0–3	0–3.5	0–4.5	0–5	0–5.5	0–6.5
	*Score 10*	>3	>3.5	>4.5	>5	>5.5	>6.5
	*Score 0*	>6	>7	>9	>10	>11	>13
Blue	Original	2	2	3	3	4	4
Red	Original	0	0	0	0	0	0

New BALANCE DI: New Brazilian Cardioprotective Nutritional Program Dietary Index; BALANCE: Brazilian Cardioprotective Nutritional Program.

## Data Availability

The data presented in this study are available on request from the corresponding author. The data are not publicly available because DICA-NUTS trial is not published yet.

## Supplementary Materials

The following supporting information can be downloaded at: https://www.mdpi.com/article/10.3390/nu14071378/s1. Table S1: BALANCE food group composition; Table S2: BALANCE caloric ranges and recommended intake of food groups; Table S3: Scoring criteria for the BALANCE DI; Table S4: AHA Dietary Targets and Healthy Diet Score for Defining Cardiovascular Health; Table S5: Foods items from the FFQ and mean cup for the scoring system for AHA-DS components measured in cups/day; Table S6: Foods from the FFQ for the scoring system for AHA-DS components measured in g/week, oz/week, and oz-equivalent servings/day; Table S7: Pearson correlations and mean differences between the AHA-DS and the BALANCE DI; Table S8: Weighted kappa on quintile rankings of agreement between the BALANCE DI, the primary and the total AHA-DS; Table S9: Pearson correlations and mean differences between the AHA-DS and the New BALANCE DI; Table S10: Weighted kappa on quintile rankings of agreement between the New BALANCE DI, the primary and the total AHA-DS. See [12,17,18,20,34].
