# Supplementary material for: Absolute and Relative Agreement between the Current and Modified Brazilian Cardioprotective Nutritional Program Dietary Index (BALANCE DI) and the American Heart Association Healthy Diet Score (AHA-DS) in Post Myocardial Infarction Patients
Source: Nutrients. 2022 Mar 25;14(7):1378. doi: 10.3390/nu14071378 (PMC9002536; doi:10.3390/nu14071378)
Supplement: Supplementary file 1 [file nutrients-14-01378-s001.zip › nutrients-1613481-supplementary.pdf]

Table S1. BALANCE food group composition

Food group	Foods
Green	Vegetables, fruits, beans and legumes, low-fat milk
Yellow	Rice, bread, pasta, oats, couscous, nuts, vegetable oil
Blue	Meat, fish, cheese, eggs
Red	Ultra-processed foods

BALANCE: Brazilian Cardioprotective Nutritional Program.

Table S2. BALANCE caloric ranges and recommended intake of food groups

Caloric range (Kcal)	Recommended intake		
	Green	Yellow	Blue
1,400	9	6	2
1,600	11	7	2
1,800	11	9	3
2,000	12	10	3
2,200	14	11	4
2,400	16	13	4

BALANCE: Brazilian Cardioprotective Nutritional Program. Adapted from Marcadenti et al, 2021 [1].

Table S3. Scoring criteria for the BALANCE DI

Index component (BALANCE food group)	Criteria for 0 point	Criteria for 10 points
Green	0	≥ the recommendation
Yellow	50% > or < the recommendation	= the recommendation
Blue	≥ 2 beyond the recommended	≤ the recommendation
Red	≥ 4	≤ 0
**Total score (range)**	**0**	**40**

BALANCE DI: Brazilian Cardioprotective Nutritional Program Dietary Index; BALANCE: Brazilian Cardioprotective Nutritional Program. Adapted from Tereza da Silva et al, 2018 [2].

Table S4. AHA Dietary Targets and Healthy Diet Score for Defining Cardiovascular Health

	**AHA Target**	**Consumption Range for Alternative Healthy Diet Score**	**Alternative Scoring Range**
**Primary dietary metrics**			
Fruits and vegetables	≥4.5 cups/d	0 to ≥4.5 cups/d	0–10
Fish and shellfish	2 or more 3.5-oz servings/wk (≥200 g/wk)	0 to ≥7 oz/wk	0–10
Sodium	≤1500 mg/d	≤1500 to >4500 mg/d	10–0
Sugar-sweetened beverages.	≤36 fl oz/wk	≤36 to >210 fl oz/wk	10–0
Whole grains	3 or more 1-oz-equivalent servings/d	0 to ≥3 oz/d	0–10
**Secondary dietary metrics**			
Nuts, seeds, and legumes	≥4 servings/wk (nuts/seeds: 1 oz; legumes: 1/2 cup)	0 to ≥4 servings/d	0–10
Processed meats	2 or fewer 1.75-oz servings/wk (≤100 g/wk)	≤3.5 to >17.5 oz/wk	10–0
Saturated fat	≤7% energy	≤7 to >15 (% energy)	10–0
AHA-DS (primary)	Ideal: 4 or 5 dietary targets (≥80%)<br>Intermediate: 2 or 3 dietary targets (40%–79%)<br>Poor: <2 dietary targets (<40%)	Sum of scores for primary metrics	0 (worst) to 100 (best)<br>Ideal: 80–100<br>Intermediate: 40–79<br>Poor: <40
AHA-DS (secondary)	Ideal: 4 or 5 dietary targets (≥80%)<br>Intermediate: 2 or 3 dietary targets (40%–79%)<br>Poor: <2 dietary targets (<40%)	Sum of scores for primary and secondary metrics	0 (worst) to 100 (best)<br>Ideal: 80–100<br>Intermediate: 40–79<br>Poor: <40

AHA-DS: American Heart Association diet score; wk: week; oz: ounces; d: day. Adapted from Virani et al, 2020 [3].

Table S5. Foods items from the FFQ and mean cup for the scoring system for AHA-DS components measured in cups/day.

AHA-DS component	FFQ item	Mean cup**
Nuts, seeds, and legumes	Beans (carioca, purple, black, green) (g)<br>Lentil, meatless peas (g)<br>Chickpeas, soybeans (g)<br>Brazilian black beans stew, bean stew (20% from total consumption)* (g)	240<br>179<br>168<br>246

*Considering a traditional recipe extracted from the Brazilian table TACO[4] (*Tabela Brasileira de Composição de Alimentos*) we consider 20% of the consumption as legumes; **United States Department of Agriculture (USDA)[5]. FFQ: food frequency questionnaire; AHA-DS: American Heart Association Diet Score.

Table S6. Foods from the FFQ for the scoring system for AHA-DS components measured in g/week, oz/week, and oz-equivalent servings/day

AHA-DS component (measure)	FFQ item
Fish and shellfish (g/week)	Fish (boiled, fried, roasted) and seafood
SSBs (oz/week)	Industrialized juice<br>Soda (common, diet, light)<br>Chocolate-flavored beverage mix (mixed with milk)
Processed meats (g/week)	Jerked meat<br>Bacon and Cold cuts (ham/mortadella/salami/frankfurter)<br>Burger, nuggets, meatball (industrialized)<br>Brazilian black beans stew, bean stew (10% from total consumption)*<br>Sandwich (hot dog, hamburger) (67% from total consumption)**

* Considering a traditional recipe extracted from the Brazilian table TACO[4] (*Tabela Brasileira de Composição de Alimentos*) we consider 10% of the consumption as processed meats; ** Considering the meat content of hot dogs and hamburger according to USDA[5] Food Central data. FFQ: food frequency questionnaire; AHA-DS: American Heart Association Diet Score; SSB: sugar sweetened beverages.

Table S7. Pearson correlations and mean differences between the AHA-DS and the BALANCE DI (n=473)

**AHA-DS**	**BALANCE DI**		
	**Pearson's correlation coefficient**	**p-value**	**Mean difference (percent [95% CI])**
Primary	0.28	<0.0001	16 (14 - 18)
Total	0.46	<0.0001	8 (7-10)

AHA-DS: American Heart Association Diet Score; BALANCE DI: Brazilian Cardioprotective Nutritional Program Dietary Index.

Table S8. Weighted kappa on quintile rankings of agreement between the BALANCE DI, the primary and the total AHA-DS

AHA-DS		BALANCE DI Q1 n(%)	Q2 n(%)	Q3 n(%)	Q4 n(%)	Q5 n(%)	Total
Primary AHA-DS	Q1 n(%)	28(30)	22(23)	18(19)	13(14)	13(14)	94(100)
	Q2 n(%)	25(26)	22(23)	17(18)	20(21)	11(12)	95(100)
	Q3 n(%)	15(16)	21(22)	17(18)	24(25)	18(19)	95(100)
	Q4 n(%)	15(16)	18(19)	23(24)	15(16)	24(25)	95(100)
	Q5 n(%)	11(12)	12(13)	20(21)	23(24)	28(30)	94(100)
	Total	94(100)	95(100)	95(100)	95(100)	94(100)	
Total AHA-DS	Q1 n(%)	40(43)	23(24)	17(18)	12(13)	2(2)	94(100)
	Q2 n(%)	22(23)	23(24)	21(22)	13(14)	16(17)	95(100)
	Q3 n(%)	17(18)	18(19)	17(18)	25(26)	18(19)	95(100)
	Q4 n(%)	10(11)	24(25)	18(19)	21(22)	22(23)	95(100)
	Q5 n(%)	5(5)	7(8)	22(23)	24(25)	36(38)	94(100)
	Total	94(100)	95(100)	95(100)	95(100)	94(100)	

BALANCE DI: Brazilian Cardioprotective Nutritional Program Dietary Index; AHA-DS: American Heart Association Diet Score.

Table S9. Pearson correlations and mean differences between the AHA-DS and the New BALANCE DI (n=473)

AHA-DS	New BALANCE DI		
	**Pearson's correlation coefficient**	**p-value**	**Mean difference (percent [95% CI])**
Primary	0.59	<0.0001	16 (14 – 18)
Secondary	0.64	<0.0001	8 (7 – 10)

AHA-DS: American Heart Association Diet Score; New BALANCE DI: New Brazilian Cardioprotective Diet Dietary Index.

Table S10. Weighted kappa on quintile rankings of agreement between the New BALANCE DI, the primary and the total AHA-DS

AHA-DS		New BALANCE DI Q1 n(%)	Q2 n(%)	Q3 n(%)	Q4 n(%)	Q5 n(%)	Total
Primary AHA-DS	Q1 n(%)	43(46)	24(25)	15(16)	10(11)	2(2)	94(100)
	Q2 n(%)	33(35)	22(23)	23(24)	15(16)	2(2)	95(100)
	Q3 n(%)	15(16)	20(21)	24(25)	23(24)	13(14)	95(100)
	Q4 n(%)	4(4)	17(18)	19(20)	29(31)	26(27)	95(100)
	Q5 n(%)	1(1)	10(11)	14(15)	18(20)	51(54)	94(100)
	Total	96(100)	93(100)	95(100)	95(100)	94(100)	
Total AHA-DS	Q1 n(%)	49(52)	26(28)	14(15)	4(4)	1(1)	94(100)
	Q2 n(%)	24(25)	24(26)	24(25)	17(18)	6(6)	95(100)
	Q3 n(%)	14(15)	19(20)	29(30)	25(26)	8(8)	95(100)
	Q4 n(%)	8(8)	16(17)	17(18)	23(24)	31(33)	95(100)
	Q5 n(%)	1(1)	8(8)	11(12)	26(28)	48(51)	94(100)
	Total	96(100)	93(100)	95(100)	95(100)	94(100)	

BALANCE DI: Brazilian Cardioprotective Nutritional Program Dietary Index; AHA-DS: American Heart Association Diet Score.

References

1. Marcadenti, A.; Weber, B.; Bersch-Ferreira, A. C.; Machado, R.; Torreglosa, C. R.; de Sousa Lara, E. M.; da Silva, L. R.; Santos, R.; Miyada, D.; Sady, E.; et al. Effects of a Brazilian cardioprotective diet and nuts on cardiometabolic parameters after myocardial infarction: study protocol for a randomized controlled clinical trial. *Trials* **2021**, 22, 582; DOI: 10.1186/s13063-021-05494-0.

2. Tereza da Silva, J.; Bersch-Ferreira, Â.C.; Torreglosa, C.R.; Weber, B.; Levy, R.B. Development of a dietary index based on the Brazilian Cardioprotective Nutritional Program (BALANCE). *Nutr. J.* **2018**, 4, 17-49; DOI: 10.1186/s12937-018-0359-5.

3. Virani, S. S.; Alonso, A.; Benjamin, E. J.; Bittencourt, M. S.; Callaway, C. W.; Carson, A. P.; Chamberlain, A. M.; Chang, A. R.; Cheng, S.; Delling, F. N.; et al. Heart Disease and Stroke Statistics-2020 Update: A Report From the American Heart Association. *Circulation* **2020**, 141, e139–e596; DOI: 10.1161/CIR.0000000000000757.

4. Tabela Brasileira de Composição de Alimentos. 4ª ed. rev. e ampl. Campinas: NEPA-UNICAMP, **2011**.

5. United States Department Of Agriculture (USDA). Available online: http://www.usda.gov. (Assessed on 01/11/2021).
